# Accidental Diagnosis of Isolated Persistent Left Superior Vena Cava After an Elective Central Venous Access Procedure for Chronic Hemodialysis: Clinical Implications and Precautions From a Case Report

**DOI:** 10.7759/cureus.44212

**Published:** 2023-08-27

**Authors:** Miguel T Coimbra, Beatriz Braga, Adriana Silva, Fernanda Sousa, José Queirós

**Affiliations:** 1 Nephrology, Hospital do Espírito Santo de Évora E.P.E., Évora, PRT; 2 Nephrology, Centro Hospitalar Universitário do Porto, Porto, PRT; 3 Radiology, Centro Hospitalar Universitário do Porto, Porto, PRT

**Keywords:** computed tomography angiography (cta), chronic hemodialysis, chronic kidney disease (ckd), central venous catheter (cvc), persistent left superior vena cava (plsvc)

## Abstract

Persistent left superior vena cava (PLSVC) is the most frequent thoracic venous anatomical variant in the general population. Isolated PLSVC, without formation of the right superior vena cava, is described in 10% of cases of PLSVC only. While it can be associated with congenital heart disease, arrhythmias, and premature death, adult patients with PLSVC are mostly asymptomatic, and the diagnosis is usually accidental. We present the case of a 72-year-old male with end-stage renal disease who was started on urgent hemodialysis through a temporary non-tunneled femoral central venous catheter (CVC) in the SLED (slow low-efficiency dialysis) modality and later remained dependent on hemodialysis. At this stage, placement of a tunneled CVC in the right internal jugular vein was necessary and fluoroscopy guidance was not available. There were no complications during the procedure, but postoperative conventional chest radiography revealed an inadequate positioning of the CVC tip in the left hemithorax, crossing the midline. Subsequently, the diagnosis of PLSVC was obtained by performing a thoracic angio-CT scan, confirming CVC tip positioning inside the PLSVC, and also excluded the presence of cardiac defects or additional anatomical variations of the great vessels of the thorax. Early evaluation for the creation of autologous vascular access was started under our care, and there were no mechanical or other complications associated with hemodialysis sessions during early follow-up after discharge.

## Introduction

Persistent left superior vena cava (PLSVC), albeit infrequent in the general population (approximately 0.3%) [1-3], is the most prevalent thoracic venous anatomical variant currently known [4].

During embryonic development, the superior vena cava (SVC) and inferior vena cava are formed during the eighth week of gestation from two primitive cardinal veins, which are part of the duct of Cuvier, and drain into the sinus venosus [5]. A PLSVC derives not only from regression failure of the left common cardinal vein, but also from the persistence of the caudal portion of the right superior cardinal vein [6]. However, if the left common cardinal vein persists, and the caudal portion of the right superior cardinal vein regresses later in intrauterine life, the left superior vena cava (LSVC) persists as an isolated PLSVC, with failure in the formation of the right superior vena cava (RSVC) [5]. This has been described in only approximately 10% of cases of PLSVC, since in the other 90%, there is a duplication of the SVC, i.e., a concomitantly persistent RSVC and LSVC [7]. A variable number of “communicating” veins between them (venous plexus of transverse veins) are present in approximately 35% of cases, which may or may not persist into the postnatal period [8].

In individuals with PLSVC, a heterotaxy syndrome (situs inversus or situs ambiguus) can be present in around 45-70% of cases [9]. Furthermore, roughly 80-90% of PLSVC cases drain into the right atrium (RA) through the coronary sinus, while only 10-20% drain into the left atrium (LA) [4,10,11]. PLSVC has been associated with congenital cardiac anomalies, cyanosis, arrhythmias, miscarriage, and premature neonatal death in the literature [9,12]. Nonetheless, while most case reports of PLSVC in the current literature have been described in pediatric patients, adult patients with PLSVC are mostly asymptomatic, and the diagnosis is frequently accidental [13].

The aim of this case is to report our experience highlighting the challenges and potential complications of central venous catheter (CVC) placement in patients starting hemodialysis treatment in the presence of an anatomic anomaly of thoracic large vessels, more specifically, an isolated PLSVC.

## Case presentation

In this report, we present a 72-year-old male, with a known history of diabetic nephropathy (ESRD) and neuropathy, peripheral obstructive artery disease (category IV in Rutherford classification [14]), and previous history of diabetic ulcers. He also had chronic heart failure (NYHA class II) and stable angina, with multivessel coronary artery disease. Recently, he developed progressively worsening symptoms of shortness of breath, chest pain, tiredness, peripheral edema, and oligoanuria within two days. Upon admission, the initial work-up revealed a previously known sinus bradycardia without de novo segmental changes in the ST segment on electrocardiography (ECG), and the transthoracic echocardiogram showed a left ventricular ejection fraction (LVEF) of 20-25%, an inferior vena cava diameter over 24 mm with less than 50% collapsibility (during inspiration), and a moderate to severe functional insufficiency of the mitral and tricuspid valves (in the acute context of hypervolemia). Chest conventional radiograph showed pulmonary edema. Blood tests revealed elevated serum creatinine levels (5.8 mg/dL) from baseline (4.6 mg/dL), hypoxemia without hyperlacticaemia, metabolic acidosis (HCO3- serum levels of 15 mEq/L in an arterial sample), and high Troponin T serum levels (maximum of 3.3 ng/mL). He was diagnosed with acute pulmonary edema with severe left ventricle dysfunction, a type 2 non-ST elevation myocardial infarction, and acute renal failure (cardiorenal syndrome type 1). The acute clinical onset was probably associated with an acute respiratory infection a few days prior, which was promptly treated. After being transferred to the Intermediate Coronary Care Unit (ICCU), he was started on urgent hemodialysis through a temporary non-tunneled CVC in the right femoral vein in the SLED (slow low-efficiency dialysis) modality, due to refractory congestion to intravenous furosemide and hypoxemic respiratory failure. He showed good hemodynamic tolerance to dialysis, with improvement of coronary symptoms (angina pectoris), and achieved and maintained euvolemia after several days of treatment, with regression of peripheral and pulmonary edema, alongside improvement of respiratory and cardiac symptoms. However, the patient remained dependent on dialysis and was later suggested for chronic intermittent hemodialysis and elective placement of a tunneled CVC in the right internal jugular vein (RIJV), while still under inpatient care. There was no previous history of thoracic vascular malformations. The summarized tunneled CVC insertion procedure is described in Table 1.

**Table 1:** Tunneled CVC insertion procedure (modified Seldinger technique) CVC: Central venous catheter

Procedure	Material: Central venous catheter Arrow® Cannon® II Plus Hemodialysis Catheter (Arrow International LLC, a subsidiary of Teleflex®, Morrisville, United States of America), V-tip design, 19 cm length (15 French, 2.0 mL arterial lumen, 2.2 mL venous lumen).
	Sterile preparation of the surgical site with a chlorhexidine-based solution
	Subcutaneous infiltration of local anesthesia (lidocaine at 2% of a 10mL 0.9% NaCl solution)
	Ultrasound-guided supraclavicular venous puncture of the right internal jugular vein at the Sedillot triangle (using the GE Healthcare® Ultrasound, LOGIQ P9 XDclear™ with a sterile ultrasound probe cover)
	Vein cannulation and guidewire introduction to prepare for vein dilation
	Insertion of the tip of the CVC along the guidewire and through the last introducer (detachable)
	Retrograde tunneling technique, with an incision for the exit site in the infraclavicular region, and manual assembly of arterial and venous ports to the respective catheter lumens
	Arterial and venous port testing with no manual resistance to aspiration or saline infusion of each port with a syringe
	CVC fixation with synthetic Prolene® suture (2.0)

During the procedure, no arrhythmic events were recorded using single-lead ECG monitoring. Systolic blood pressure (SBP) was persistently above 120 mmHg and below 140 mmHg, with peripheral oxygen saturation over 98%, and the patient remained asymptomatic. In the immediate postoperative period, our patient was hemodynamically stable, with no clinical or laboratory findings suggestive of active bleeding.

**Figure 1:**
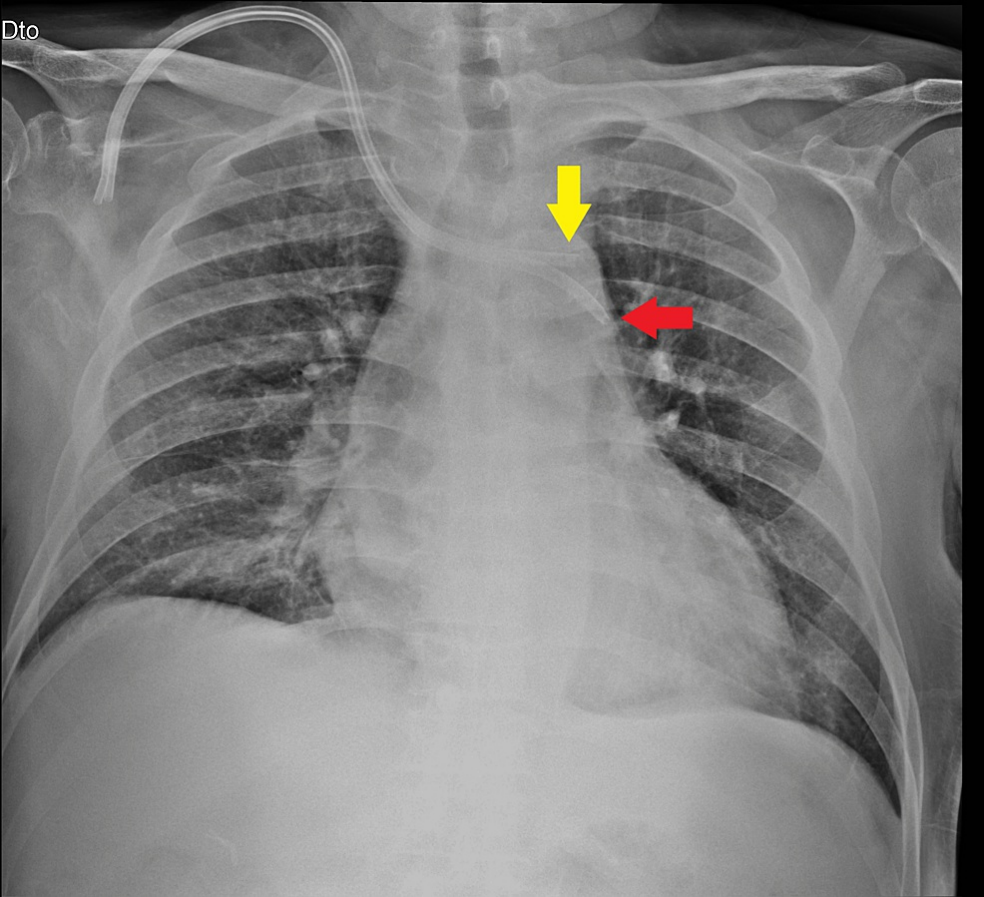
Conventional chest radiography of the CVC placement Conventional chest radiography shows placement of the double-lumen hemodialysis catheter tip in the left hemithorax, crossing the chest midline. Red arrow: Longer venous limb of the tunneled CVC; yellow arrow: shorter arterial limb. CVC: Central venous catheter

**Figure 2:**
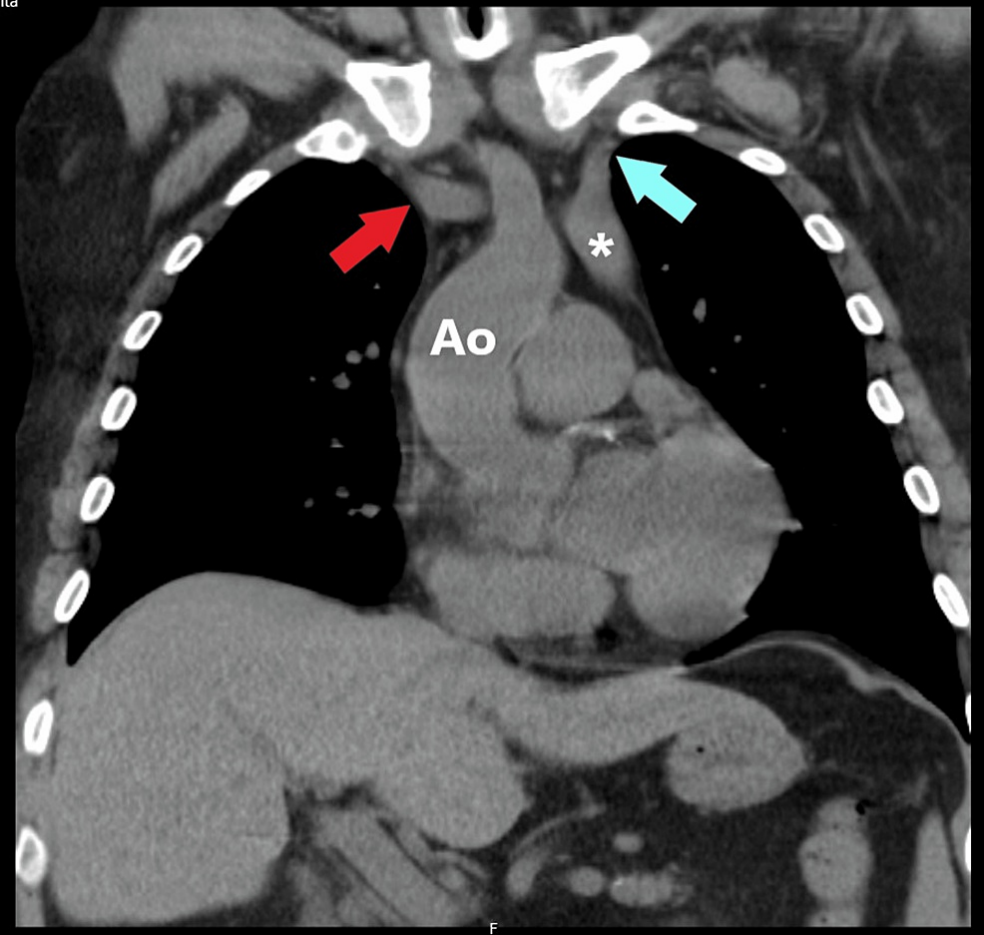
Reformatted coronal image from a chest CT scan performed five years prior to this episode. Images from the database at our institution were reviewed after the referral of this case to a radiologist. White asterisk: Left superior vena cava; red arrow: right internal jugular vein; light blue arrow: left internal jugular vein; Ao: aorta.

**Figure 3:**
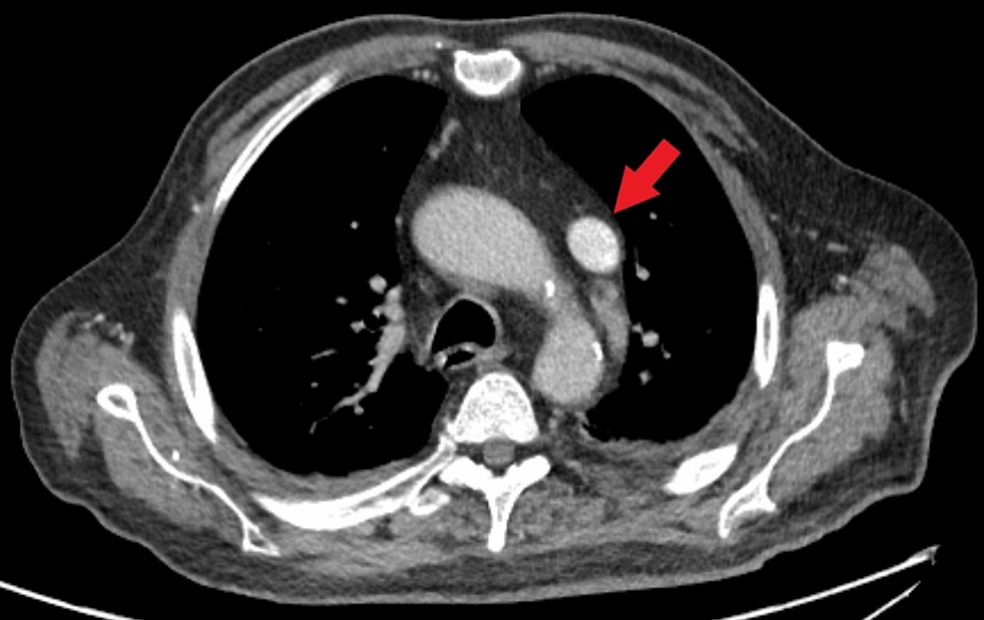
CT angiography of the chest (venous phase, transverse plane) An isolated persistent left superior vena cava is visible (red arrow). The right superior vena cava is not visible.

**Figure 4:**
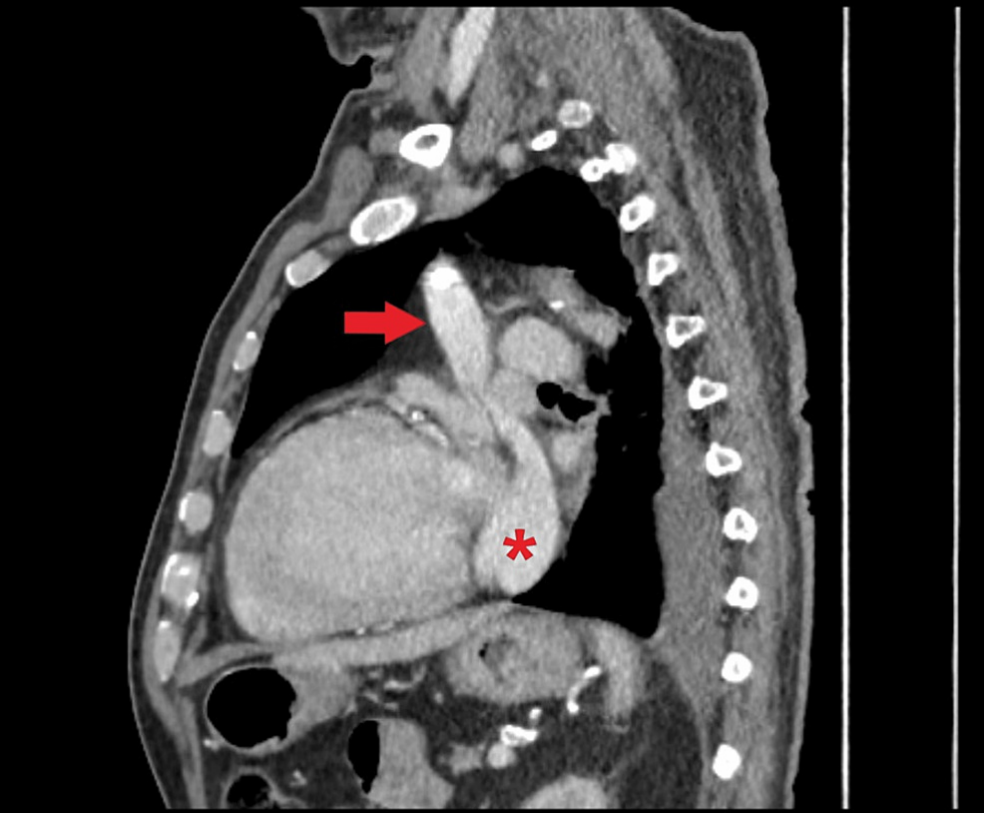
CT angiography of the chest (venous phase, sagittal plane) The left superior vena cava (red arrow) is visible draining contrast-enhanced venous blood into a dilated coronary sinus (red asterisk). The CVC tip is visible in the upper portion of the PLSVC. PLSVC: Persistent left superior vena cava; CVC: central venous catheter

**Figure 5:**
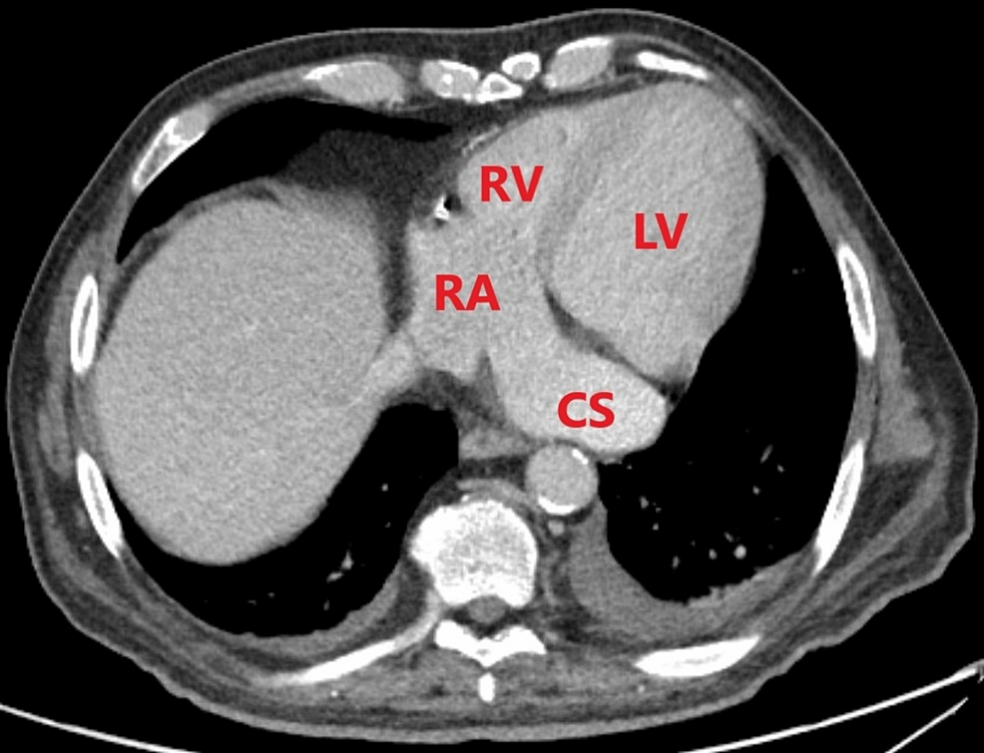
CT angiography of the chest (venous phase, transverse plane) The dilated coronary sinus (CS) drains contrast-enhanced venous blood into the right atrium (RA). The left atrium is not visible. RV: Right ventricle; LV: left ventricle.

Conventional chest radiography at the end of the tunneled CVC insertion procedure revealed inadequate positioning of the CVC tip in the left hemithorax, crossing the chest midline (Figure 1), which initially alerted us to the possibility of either CVC displacement or an anatomic variant of the thoracic venous system. Complications such as hemithorax or pneumothorax were also excluded, and there were no symptoms suggesting cardiac tamponade. Subsequently, the diagnosis of PLSVC was further suggested based on the reassessment with a radiologist of the images of a chest computed tomography (CT) scan performed five years prior to this episode (Figure 2), without contrast material, in the context of infectious respiratory complications. We requested a new CT angiography of the chest in the current episode to confirm the diagnosis of PLSVC (Figures 3, 4), which was seen draining anatomically into a dilated coronary sinus and into the right atrium (Figure 5). Chest CT angiography also excluded cardiac defects or additional anatomical variations of the great vessels of the thorax.

## Discussion

In patients with a timely diagnosis of PLSVC in its variant with agenesis of the RSVC, regarding the patient's vascular life plan, the best approach should be an early evaluation and creation of autologous vascular access before CVC placement, following National Kidney Foundation Kidney Disease Outcomes Quality Initiative (NKF-KDOQI)TM guidelines [15], even among patients with poor vascular integrity as is our case, to avoid urgent CVC placement for dialysis and improve clinical outcomes in the long term. Furthermore, PLSVC can be responsible for the induction and persistence of arrhythmia in almost 50% of these patients, who have a higher incidence of cardiac conduction anomalies, including atrial fibrillation, atrial flutter, Wolff-Parkinson-White syndrome, and atrioventricular conduction blocks [16]. The risk of cardiogenic shock is increased, and therefore, it is crucial to proceed cautiously with new invasive procedures, including new CVC insertions or pacemaker insertions [17].

We know that tunneled CVC tip positioning in the RSVC outside the right atrium is associated with fibrin sheath formation and increased risk of thrombosis and central venous stenosis [18], and current NKF-KDOQI recommendations suggest placement of the tip in the mid right atrium [15]. However, in a patient with PLSVC, catheter tip malposition into the coronary sinus is also a known complication with increased risk of thrombosis, acute coronary syndrome, and potentially fatal arrhythmia [19-21], which we believe can outweigh the risk of central venous stenosis of the PLSVC. It is important to consider that any further manipulation or replacement of the CVC initially placed in the coronary sinus also increases the risk of perforation with cardiac tamponade [22,23]. We decided to maintain the current tunneled CVC despite its suboptimal length, given its optimal function during hemodialysis sessions and the absence of associated mechanical, vascular, or infectious complications during early follow-up.

The NKF-KDOQI guidelines recommend fluoroscopy-guided placement for all hemodialysis CVCs, in order to monitor the progression of the guidewire and to allow for ideal positioning of the catheter tip in most cases [15]. However, we do not have access to an interventional radiology suite on a daily basis, and it is currently reserved, according to our internal protocol, for the placement and replacement of all tunneled CVCs in the left internal jugular vein (LIJV), or in the RIJV in specific cases associated with complications, such as central venous stenosis.

In a patient with PLSVC without readily available autologous access for hemodialysis, or if the patient’s cognitive status and comorbidities render him ineligible for chronic peritoneal dialysis, we propose preferential placement of the long-term tunneled CVC in the LIJV under fluoroscopy guidance. Contrary to the general population, the LIJV in patients with isolated PLSVC is more vertical, i.e., the angle of incidence of the LIJV in the superior vena cava in this subset is greater than the angle of the RIJV in the SVC [15], which can theoretically decrease the risk of mechanical and vascular complications associated with the CVC, such as central venous stenosis and obstruction of flow [24].

If a long-term tunneled CVC is placed in the inpatient setting following an urgent start of hemodialysis treatment, as was our case, and the patient opts for hemodialysis as the preferred renal replacement therapy modality, we favor the vascular mapping and creation of an autologous access as soon as possible, as part of the patient's vascular life plan. It should be placed preferably in the upper limb contralateral to the current CVC insertion site [25] since a CVC significantly reduces the patency of arterial-venous access created in the ipsilateral upper limb, and we understand that there is no evidence to suggest that cases of isolated PLSVC should not follow this rule.

## Conclusions

With this case report, we hope to raise awareness for an illustrated example that goes in favor of a generalized fluoroscopic control approach in the practice of long-term CVC placement for hemodialysis, as well as highlight the prognosis and the most appropriate guidance to follow in a specific case following CVC placement with an anatomical variation of the thoracic venous system.
